# Risk Score for Hepatocellular Cancer in Adults Without Viral Hepatitis or Cirrhosis

**DOI:** 10.1001/jamanetworkopen.2024.43608

**Published:** 2024-11-06

**Authors:** Ysabel C. Ilagan-Ying, Kirsha S. Gordon, Janet P. Tate, Joseph K. Lim, Jessie Torgersen, Vincent Lo Re, Amy C. Justice, Tamar H. Taddei

**Affiliations:** 1 Department of Medicine, Yale School of Medicine, New Haven, Connecticut; 2 Department of Medicine, Veterans Affairs Connecticut Healthcare, West Haven, Connecticut; 3 Division of Infectious Diseases, Department of Medicine and Center for Clinical Epidemiology and Biostatistics, Department of Biostatistics, Epidemiology, and Informatics, Perelman School of Medicine, University of Pennsylvania, Philadelphia; 4 Department of Health Policy and Management, Yale School of Public Health, New Haven, Connecticut

## Abstract

**Importance:**

Hepatocellular carcinoma (HCC) is typically detected only at advanced stages when treatment options are limited. Most of the current HCC risk models focus on patients with viral hepatitis or diagnosed cirrhosis or require variables not routinely available in clinical care.

**Objective:**

To identify modifiable HCC risk factors in the general population and to develop a risk score to inform HCC screening and risk-factor modification interventions for high-risk individuals without viral hepatitis or decompensated cirrhosis.

**Design, Setting, and Participants:**

This cohort study analyzed demographic, clinical, laboratory, and diagnostic data from the US Department of Veterans Affairs (VA) electronic health records. Data were divided into development and validation samples. Veterans aged 30 to 95 years were included, and those with hepatitis B or C virus infection, hepatic decompensation, or prevalent HCC were excluded. Patients were followed up until the occurrence of HCC diagnosis, death, or December 31, 2021. A Cox proportional hazards regression model for 10-year risk of HCC was developed and used to create an HCC risk score, and performance in development and validation samples and in patient subgroups was evaluated. One outpatient visit date per person at least 18 months after VA entry, between October 1, 2007, and March 31, 2020, was randomly selected and used as the index date for the start of follow-up. Analyses were performed from March 2023 to May 2024.

**Exposures:**

Age, sex, race and ethnicity, body mass index, liver fibrosis (detected with Fibrosis-4 Index [FIB-4]), diabetes status, smoking status, and alcohol use.

**Main Outcomes and Measures:**

First HCC diagnosis during follow-up. This information was ascertained from VA national cancer registry topography and histology codes and from *International Classification of Diseases, Ninth Revision* and *International Statistical Classification of Diseases, Tenth Revision, Clinical Modification* diagnosis codes for the inpatient or outpatient visits.

**Results:**

This study of 6 509 288 veterans included 6 048 917 males (92.9%), with a median (IQR) age of 65 (54-74) years, who identified as being of Hispanic (5.3%), non-Hispanic Black (15.0%), non-Hispanic White (68.9%), or other (4.6%) race and ethnicity. Overall, 15 142 patients (0.2%) developed HCC, 69.5% of whom had FIB-4 of 3.25 or lower at baseline. While FIB-4 was the most important variable, age, sex, race and ethnicity, body mass index, diabetes, smoking, and alcohol use were also informative. Discrimination in the development sample was better than FIB-4 alone (C statistic, 0.83 [95% CI, 0.82-0.85] vs 0.79 [95% CI, 0.77-0.80]). The HCC risk score performed consistently well in the validation sample and in all subgroups. A FIB-4 threshold of 3.25 would screen 5.0% of the cohort at a cost of 28 false-positives for every true-positive; a model risk score of 58 would screen 4.7% of the cohort at a cost of 23 false-positives for every true-positive.

**Conclusions and Relevance:**

Results of this study suggest that a multivariable risk score that uses routinely available clinical data outperforms FIB-4 alone in identifying patients at risk of HCC who do not have viral hepatitis or hepatic decompensation at baseline.

## Key Points

**Question:**

Can risk factors for hepatocellular carcinoma (HCC) among adults without viral hepatitis or decompensated cirrhosis be identified and used to develop a risk score to inform screening?

**Findings:**

In a cohort study of 6 509 288 patients in the Veterans Affairs health care system, a risk score was developed using the Fibrosis-4 Index, age, sex, race and ethnicity, body mass index, diabetes status, smoking status, and alcohol use. This HCC risk score performed consistently well in the validation sample and in all subgroups.

**Meaning:**

Findings of this study suggest that a risk score developed with routinely available clinical data can outperform Fibrosis-4 Index alone in identifying patients at risk of HCC who do not have viral hepatitis or hepatic decompensation at baseline.

## Teaser

This cohort study describes the risk factors associated with onset of hepatocellular carcinoma and a risk score designed to assess and mitigate this cancer among patients in the Veterans Affairs health care system.

## Introduction

Hepatocellular carcinoma (HCC) is a common and lethal malignant neoplasm worldwide. In the US, virally associated HCC incidence is decreasing due to effective hepatitis B virus (HBV) vaccination programs and direct-acting antiviral therapies for hepatitis C virus (HCV).^1^ Yet HCC incidence due to chronic alcohol-associated liver disease (ALD) and metabolic dysfunction–associated steatotic liver disease (MASLD) is increasing. Nearly half of HCC events in the US occur among those without viral hepatitis.^2^ Risk factors, such as obesity, diabetes, unhealthy alcohol use, and smoking, are associated with ALD, MASLD, and the overlap of MASLD and increased alcohol intake (MetALD).^3,4^

Rather than address the modifiable behavioral and metabolic precursors underlying MASLD, ALD, and MetALD, current guidelines focus on HCC screening in populations with cirrhosis (from any cause) or chronic HBV infection.^5,6^ Furthermore, estimates of unrecognized cirrhosis at HCC diagnosis are as high as 39.0%,^7^ and an estimated 38.5% of people with underlying steatotic liver disease do not have cirrhosis at the time of HCC diagnosis.^8,9^ Thus, current guidelines focusing on early HCC detection may be missing opportunities for screening, early detection, and primary prevention targeting modifiable risk factors in a high-risk population.^10^

The risk of HCC is multifactorial, and individuals often have more than 1 risk factor.^11,12^ However, specialists have focused on developing risk scores for progression of fibrosis, often relying on liver imaging or liver stiffness measurement tests, which are not readily available to primary care clinicians.^10,13^ However, once cirrhosis is present, other risk factors for HCC are eclipsed,^14^ suggesting that interventions focused on obesity, diabetes, unhealthy alcohol use, and smoking may be less effective with cirrhosis. If a subset of higher-risk individuals in the general population (without viral hepatitis or known cirrhosis) can be identified using routinely available clinical data, then expanded screening and targeted primary prevention may be cost-effective. Thus, in this study, because direct measures of steatosis and fibrosis are not readily available in the electronic health record (EHR), we aimed (1) to identify modifiable HCC risk factors in the general population, including the Fibrosis-4 Index (FIB-4),^15,16^ a clinically available and validated indicator of liver fibrosis based on aspartate aminotransferase (AST) and alanine aminotransferase (ALT) levels, platelet count, and age and (2) to develop a risk score to inform HCC screening and risk-factor modification interventions for high-risk individuals without viral hepatitis or decompensated cirrhosis.

## Methods

### Data Source

The Department of Veterans Affairs (VA) is the largest integrated health care system in the US, equipped with a centralized EHR that contains data from over 1200 care centers, including hospitals, medical centers, and outpatient clinics. We obtained information from the VA Corporate Data Warehouse, a repository of nationwide patient-level data from all VA care centers. Demographic, clinical, laboratory, and diagnostic data, including *International Classification of Diseases, Ninth Revision (ICD-9)* and *International Statistical Classification of Diseases, Tenth Revision, Clinical Modification (ICD-10-CM)* diagnosis codes, were extracted for inpatient and outpatient VA visits as well as treatments rendered outside of the VA system and covered by Medicare. Deaths were identified from the Veterans Health Administration Vital Status File, which uses data from the Social Security Administration Death Master File, Medicare Vital Status File, and Beneficiary Identification and Records Locator Subsystem. The VA Connecticut Healthcare System and Yale School of Medicine Institutional Review Boards approved this cohort study and waived the informed consent requirement because the study was based exclusively on data already collected as part of routine medical care. The study was compliant with the Health Insurance Portability and Accountability Act. We followed the Transparent Reporting of a Multivariable Prediction Model for Individual Prognosis or Diagnosis (TRIPOD) reporting guideline.

### Population

Patients aged 30 to 95 years were included. We used a 2-stage sampling design to randomly select a single index visit per patient, giving preference to primary care visits. In stage 1, we identified outpatient clinic visits that included routine blood pressure reading. For each year from 2008 to 2020, we randomly selected 1 outpatient visit date per person that was at least 18 months after the date of the first outpatient diagnosis in the EHR, selecting a primary care visit if one was available or another medical visit. We excluded patients with uncertain date of death, evidence of HBV or HCV infection by laboratory test (detectable HBV surface antigen, DNA, or genotype or detectable HCV antibody, RNA, or genotype) or by *ICD-9* or *ICD-10-CM* code (eTable 3 in Supplement 1), or a diagnosis of either hepatic decompensation (using a previously validated algorithm)^17^ or HCC (by *ICD-9* or *ICD-10-CM* code) at any time on or before the visit. For each eligible visit, we searched for data closest to the visit date for laboratory tests (18 months before to 14 days after), body mass index (BMI; up to 2 years before), and alcohol use (up to 5 years prior). In stage 2, to ensure a range of follow-up times and disease severity, we pooled visits from each year for those patients with complete covariate data. We then randomly selected a single visit per person from all eligible visits. This index visit date was used as the start of follow-up.

### Primary Outcome

The primary outcome was first HCC diagnosis during follow-up. We defined HCC in 2 ways. First, we used the VA national cancer registry, which records cancers diagnosed or treated within the VA. HCC diagnoses were identified by topography codes (C22.0, liver) and histology codes (8170-8180, HCC) from the *International Classification of Diseases for Oncology, Third Revision (ICD-O-3).* Second, we used *ICD-9* or *ICD-10-CM* codes (155.0, malignant neoplasm of liver, primary; C22.0, liver cell carcinoma; C22.8, malignant neoplasm of liver, primary, unspecified as to type), requiring 1 inpatient diagnosis or more than 1 outpatient diagnosis. Prior to *ICD-10-CM,* there was no HCC-specific code, so we used nonspecific codes. We increased specificity by requiring the absence of bile duct disease (codes 155.1, intrahepatic bile duct carcinoma; C22.1, malignant neoplasm of intrahepatic bile ducts). We chose these codes based on comparison with the VA cancer registry and extensive review of medical records.^18^

### Covariates

We chose variables associated with HCC based on those identified in the literature but routinely available and directly analyzable within EHRs. These variables included age, sex, race and ethnicity, FIB-4, diabetes status, smoking status, alcohol use, and BMI (calculated as weight in kilograms divided by height in meters squared, with a BMI of 25-29 indicating overweight and ≥30 indicating obesity). Race and ethnicity (recorded in the VA EHR from self-report) was categorized as Hispanic, non-Hispanic Black, non-Hispanic White, other (American Indian or Alaska Native, Asian, Native Hawaiian or Other Pacific Islander, and multiracial), and unknown. Given that genetic (*PNPLA3* gene) sequence variations associated with HCC are prevalent in Hispanic people but less so among White and Black individuals, it is important to include race and ethnicity in risk prediction.

Diabetes status was based on *ICD-9* or *ICD-10-CM* diagnosis codes (eTable 3 in Supplement 1). Alcohol use was classified as history of alcohol use disorder (AUD, by *ICD-9* or *ICD-10-CM* codes) or, in the absence of AUD, was assessed with Alcohol Use Disorders Identification Test–Consumption (AUDIT-C) and categorized as abstinent (score of 0), low risk (score of 1-2 [females] or 1-3 [males]), moderate risk (score of 3-7 [females] or 4-7 [males]), or high risk (score of ≥8 [females and males]).^19,20^ Smoking status was ascertained using a previously validated algorithm that categorizes smoking status as never, current, or former.^21^

FIB-4 is a readily available clinical index derived initially in persons with HIV-HCV co-infection from a composite of AST and ALT levels, platelet count, and age: [FIB-4 = (Age × AST) / (Platelet count × √ALT)].^15^ Standard FIB-4 categories in a general medical population are lower than 1.45 (95% negative predictive value to exclude substantial fibrosis with high sensitivity), 1.45 to 3.25 (moderate fibrosis risk), and higher than 3.25 (82% positive predictive value [PPV] to confirm advanced fibrosis or cirrhosis with high specificity).^22^ FIB-4 has been validated with varying diagnostic accuracy in populations with viral, alcohol-associated, and steatotic liver diseases.^15,16,22^

We sampled only from visits with complete data (eFigure 1 in Supplement 1). Overall, 81% of visits had complete data (FIB-4, smoking, alcohol, and BMI), and 92% of eligible patients had at least 1 visit with complete data. FIB-4 components were from the same day for 80% of visits.

### Statistical Analysis

We split the sample into development (index visits for 2008 to 2011 and 2015 to 2020) and validation (index visits for 2012 to 2014) samples. We tabulated summary statistics separately for the full sample, development sample, and validation sample. Individuals were followed up from the index date until the earliest occurrence of HCC event, death, December 31, 2021, or 6 months after the last VA or Medicare (whichever was later) diagnosis in any setting. We calculated HCC incidence rates per 1000 person-years according to patient characteristics, stratified by FIB-4 level (<1.45, 1.45-3.25, >3.25).

We created a Cox proportional hazards regression model to evaluate associations with 10-year risk of HCC in the development sample. For ease of interpretation, we created a model in which all variables were categorical, with fine gradations; for maximal discrimination, we created another model treating all variables as continuous and decomposing FIB-4 into its continuous components. We compared the multivariable model to FIB-4 alone (categorical and continuous). Because of the large sample size, we measured discrimination (Harrell C statistic) on 10 random samples of 500 000 and averaged the results. We used a χ^2^ test to assess the relative importance of each risk factor. Analyses were performed from March 2023 to May 2024, using SAS, version 9.4 (SAS Institute Inc). Statistical significance was defined as 2-tailed *P* < .05.

For implementation of the model, we translated regression output into a single risk score used for subsequent analyses. For each variable, we multiplied regression coefficients by each patient’s individual values and then summed to create a linear predictor (Xbeta in SAS). Next, we scaled to 0 to 100 by dividing each patient’s Xbeta by the difference of highest and lowest values across all patients in the development sample. We estimated incidence by using the risk score in a Cox proportional hazards regression model. Observed event rates as a function of score were estimated using the Kaplan-Meier method. For each 5-point interval of score, we calculated HCC incidence with 95% CI. We compared plots of HCC incidence with HCC risk score at 1, 5, and 10 years in the development and validation samples. Satisfied with the concordance, we used the full sample to assess equality of performance (fairness) in subgroups (age <65 years or ≥65 years; females or males; Hispanic, non-Hispanic Black, or non-Hispanic White; FIB-4 level; diabetes status; smoking status; alcohol use; and BMI). We compared performance characteristics of the HCC risk score with those of FIB-4 threshold of 3.25.^23^

We conducted 4 sensitivity analyses. Because the extent of missing AUDIT-C scores was much higher in 2008 than in other years, we reran the risk score model, excluding index visits in 2008. We also reran the model that restricted to AUDIT-C scores within 1 year of the index visit and to BMI within 1 year to account for any missingness more proximate to the baseline visit. Additionally, we expanded the outcome to include malignant neoplasm of liver, which was not specified as primary or secondary (*ICD-9* or *ICD-10-CM* codes 155.2 and C22.9) and other specified carcinomas of liver (*ICD-10-CM* code C22.7).

## Results

There were 11.7 million US veterans alive as of October 1, 2007, who had any VA outpatient diagnosis by September 30, 2018. Of these patients, 7 893 126 received outpatient medical care from 2008 to 2020 (eFigure 1 in Supplement 1). After excluding those with HBV or HCV and those with hepatic decompensation or HCC diagnosed prior to the visit, there were 7 453 532 eligible veterans, 91.6% of whom had complete covariate data. In preliminary analysis, we found insufficient HCC events in those under age 30 years or over age 95 years; thus, these categories were excluded.

We identified 6 509 288 veterans, including 460 371 females (7.1%) and 6 048 917 males (92.9%), with a median (IQR) age of 65 (54-74) years, and who identified having Hispanic (5.3%), non-Hispanic Black (15.0%), non-Hispanic White (68.9%), or other (4.6%) race and ethnicity (Table 1). Of the full sample, 55.8% had FIB-4 lower than 1.45, and 5.1% had FIB-4 higher than 3.25. The development sample comprised 5 119 775 patients (2008-2011 and 2015-2020), and the validation sample consisted of 1 389 513 patients (2012-2014). More than half (53.6%) of the randomly selected index dates were from 2015 to 2020. The development and validation datasets were similar in characteristics.

**Table 1.** Baseline Characteristics of Veterans at Randomly Selected Index Visit From 2008 to 2020

Characteristic	Veterans, No. (%)		
	Full sample (N = 6 509 288)	Development (n = 5 119 775	Validation (n = 1 389 513)
Index visit, y			
2008-2011	1 632 984 (25.1)	1 632 984 (31.9)	NA
2012-2014	1 389 513 (21.3)	NA	1 389 513 (100.0)
2015-2020	3 486 791 (53.6)	3 486 791 (68.1)	NA
Age, y			
Median (IQR)	65 (54-74)	65 (53-74)	65 (55-75)
<50	1 247 842 (19.2)	1 016 460 (19.9)	231 382 (16.7)
50-64	1 841 450 (28.3)	1 424 884 (27.8)	416 566 (30.0)
65-79	2 388 382 (36.7)	1 893 441 (37.0)	494 941 (35.6)
≥80	1 031 614 (15.8)	784 990 (15.3)	246 624 (17.7)
Sex			
Female	460 371 (7.1)	374 306 (7.3)	86 065 (6.2)
Male	6 048 917 (92.9)	4 745 469 (92.7)	1 303 448 (93.8)
Race and ethnicity^a^			
Black, non-Hispanic	979 291 (15.0)	776 762 (15.2)	202 529 (14.6)
Hispanic	341 995 (5.3)	276 082 (5.4)	65 913 (4.7)
White, non-Hispanic	4 482 953 (68.9)	3 510 365 (68.6)	972 588 (70.0)
Other^b^	298 109 (4.6)	234 858 (4.6)	63 251 (4.6)
Unknown	406 940 (6.3)	321 708 (6.3)	85 232 (6.1)
FIB-4 threshold			
<1.45	3 632 895 (55.8)	2 904 533 (56.7)	728 362 (52.4)
1.45-3.25	2 547 170 (39.1)	1 967 982 (38.4)	579 188 (41.7)
>3.25	329 223 (5.1)	247 260 (4.8)	81 963 (5.9)
Diabetes status	1 147 638 (17.6)	778 121 (15.2)	369 517 (26.6)
Smoking status			
Never	2 187 497 (33.6)	1 736 434 (33.9)	451 063 (32.5)
Current	2 039 900 (31.3)	1 591 570 (31.1)	448 330 (32.3)
Former	2 281 891 (35.1)	1 791 771 (35.0)	490 120 (35.3)
Alcohol use^c^			
AUD	895 794 (13.8)	702 579 (13.7)	193 215 (13.9)
Abstinent	2 818 395 (43.3)	620 (42.9)	620 775 (44.7)
Lower risk	2 150 318 (33.0)	1 706 238 (33.3)	444 080 (32.0)
Moderate risk	581 582 (8.9)	463 304 (9.0)	118 278 (8.5)
High risk	63 199 (1.0)	50 034 (1.0)	13 165 (0.9))
BMI			
<20	179 211 (2.8)	138 195 (2.7)	41 016 (3.0)
20 to <25	1 199 567 (18.4)	931 220 (18.2)	268 347 (19.3)
25 to <30	2 378 779 (36.5)	1 865 307 (36.4)	513 472 (37.0)
30 to <35	1 679 885 (25.8)	1 330 203 (26.0)	349 682 (25.2)
35 to <40	709 977 (10.9)	566 085 (11.1)	143 892 (10.4)
≥40	361 869 (5.6)	288 765 (5.6)	73 104 (5.3)
Follow-up, y			
Median (IQR)	4.5 (2.5-7.6)	3.9 (2.4-6.3)	7.8 (4.5-8.8)
<5	3 584 674 (55.1)	3 209 774 (62.7)	374 900 (27.0)
5 to <10	2 151 706 (33.1)	1 184 420 (23.1)	967 286 (69.6)
≥10	772 908 (11.9)	725 581 (14.2)	47 327 (3.4)
Events during maximum 10-y follow-up			
HCC	15 142 (0.2)	10 896 (0.2)	4246 (0.3)
Death	1 873 819 (28.8)	1 354 592 (26.5)	519 227 (37.4)

Abbreviations: AUD, alcohol use disorder; BMI, body mass index (calculated as weight in kilograms divided by height in meters squared; ≥30 indicates obesity and ≥25 indicates overweight); FIB-4, Fibrosis-4 Index; HCC, hepatocellular carcinoma; NA, not applicable.

^a^ Race and ethnicity were self-reported and obtained from the US Department of Veterans Affairs electronic health record.

^b^ Other includes American Indian or Alaska Native, Asian, Native Hawaiian or Other Pacific Islander, and multiracial.

^c^ Alcohol use was classified as history of AUD or, in the absence of AUD, was assessed with Alcohol Use Disorders Identification Test–Consumption and categorized as abstinent (score of 0), low risk (score of 1-2 [females] or 1-3 [males]), moderate risk (score of 3-7 [females] or 4-7 [males]), or high risk (score of ≥8 [females and males]).

Diabetes was less common in the development dataset than the validation dataset (15.2% vs 26.6%). Never, current, and former smokers represented nearly equal thirds of the cohort. Between the development and validation samples, AUD was diagnosed in 13.7% and 13.9% of patients, abstinence was reported in 42.9% and 44.7%, moderate-risk drinking in 9.0% and 8.5%, and high-risk drinking in 1.0% and 0.9%. Overall, most patients had overweight or obesity, and only 2.8% of patients had a BMI less than 20.

Median (IQR) follow-up time was 3.9 (2.4-6.3) years in the development dataset and 7.8 (4.5-8.8) years in the validation dataset. In the development sample, 14.2% of individuals had 10 or more years of follow-up. During a maximum of 10 years of follow-up, there were 15 142 HCC events (0.2%) and 1 873 819 deaths (28.8%) overall. A total of 15 142 patients (0.2%) developed HCC, of whom 10 519 (69.5%) had FIB-4 of 3.25 or lower at baseline.

Incidence of HCC varied by FIB-4 level, ranging from 0.17 events per 1000 person-years in those with FIB-4 lower than 1.45 to 3.6 per 1000 person-years in those with FIB-4 higher than 3.25 (Table 2). The absolute number of HCC cases was greater in those with FIB-4 of 1.45 to 3.25 (7253 events) than in those with FIB-4 higher than 3.25 (4623 events). Variation of risk within FIB-4 strata was observed by levels of other risk factors. For example, among patients with FIB-4 higher than 3.25, incidence of HCC per 1000 person-years was 2.1 in those younger than 50 years, 7.2 in those aged 50 to 64 years, 5.0 in those aged 65 to 79 years, and 1.3 in those aged 80 years or older. FIB-4 higher than 3.25 was more common in non-Hispanic White (238 031 of 4 482 953 [5.3%]) individuals than non-Hispanic Black (31 433 of 979 291 [3.2%]) or Hispanic (12 720 of 341 995 [3.7%]) people. However, HCC rates among those with FIB-4 higher than 3.25 were higher in Hispanic patients (9.2 per 1000 person-years) and lower in Black patients (1.7 per 1000 person-years) compared with White patients (3.6 per 1000 person-years).

**Table 2.** Bivariate Associations of Risk Factors With Incident HCC Rate During a Maximum 10-Year Follow-Up, Stratified by Baseline FIB-4 Index Level From 2008 to 2020

Risk factor	Baseline FIB-4 threshold among veterans (N = 6 509 288)								
	<1.45			1.45-3.25			>3.25		
	No. (%)	HCC cases, No. (%)	Rate per 1000 person-years	No. (%)	HCC cases, No. (%)	Rate per 1000 person-years	No. (%)	HCC cases, No. (%)	Rate per 1000 person-years
Total	3 632 895 (55.8)	3266 (21.6)	0.17	2 547 170 (39.1)	7253 (47.9)	0.57	329 223 (5.1)	4623 (30.5)	3.6
Age, y									
<50	1 187 233 (32.7)	161 (4.9)	0.03	55 284 (2.2)	54 (0.7)	0.17	5325 (1.6)	51 (1.1)	2.1
50-64	1 324 681 (36.5)	1166 (35.7)	0.15	477 774 (18.8)	1553 (21.4)	0.54	38 995 (11.8)	1292 (27.9)	7.2
65-79	975 414 (26.8)	1701 (52.1)	0.36	1 296 340 (50.9)	4121 (56.8)	0.62	116 628 (35.4)	2524 (54.6)	5.0
≥80	145 567 (4.0)	238 (7.3)	0.44	717 772 (28.2)	1525 (21.0)	0.53	168 275 (51.1)	756 (16.4)	1.3
Sex									
Female	390 705 (10.8)	96 (2.9)	0.05	64 305 (2.5)	68 (0.9)	0.20	5361 (1.6)	38 (0.8)	1.7
Male	3 242 190 (89.2)	3170 (97.1)	0.19	2 482 865 (97.5)	7185 (99.1)	0.58	323 862 (98.4)	4585 (99.2)	3.6
Race and ethnicity^a^									
Black, non-Hispanic	659 270 (18.1)	371 (11.4)	0.10	288 588 (11.3)	449 (6.2)	0.31	31 433 (9.5)	213 (4.6)	1.7
Hispanic	223 359 (6.1)	183 (5.6)	0.16	105 916 (4.2)	517 (7.1)	0.96	12 720 (3.9)	451 (9.8)	9.2
White, non-Hispanic	2 392 872 (65.9)	2358 (72.2)	0.19	1 852 050 (72.7)	5354 (73.8)	0.58	238 031 (72.3)	3395 (73.4)	3.6
Other^b^	179 776 (4.9)	162 (5.0)	0.17	104 131 (4.1)	338 (4.7)	0.64	14 202 (4.3)	230 (5.0)	4.0
Unknown	177 618 (4.9)	192 (5.9)	0.22	196 485 (7.7)	595 (8.2)	0.66	32 837 (10.0)	334 (7.2)	2.9
Diabetes status									
No	3 100 913 (85.4)	1888 (57.8)	0.12	2 006 738 (78.8)	3839 (59.2)	0.39	253 999 (77.2)	2453 (53.1)	2.5
Yes	531 982 (14.6)	1378 (42.2)	0.42	540 432 (21.2)	3414 (47.1)	1.17	75 224 (22.8)	2170 (46.9)	7.3
Smoking status									
Never	1 230 682 (33.9)	703 (21.5)	0.11	843 691 (33.1)	1815 (25.0)	0.42	113 124 (34.4)	1338 (28.9)	2.9
Current	1 370 970 (37.7)	1397 (42.8)	0.20	599 208 (23.5)	2187 (30.2)	0.76	69 722 (21.2)	1312 (28.4)	5.1
Former	1 031 243 (28.4)	1166 (35.7)	0.21	1 104 271 (43.4)	3251 (44.8)	0.59	146 377 (44.5)	1973 (42.7)	3.4
Alcohol use^c^									
AUD	549 746 (15.1	613 (18.8)	0.22	296 618 (11.6)	1 266 (17.5)	0.90	49 430 (15.0)	1 228 (26.6)	6.4
Abstinent	1 399 958 (38.5)	1 487 (45.5)	0.20	1 245 627 (48.9)	3 492 (48.1)	0.59	172 810 (52.5)	1 996(43.2)	3.2
Lower risk	1 317 084 (36.3)	924 (28.3)	0.13	755 996 (29.7)	1 895 (26.1)	0.47	77 238(23.5)	995 (21.5)	3.0
Moderate risk	323 718 (8.9)	218 (6.7)	0.12	230 526 (9.1)	526 (7.3)	0.42	27 338 (8.3)	342 (7.4)	2.8
High risk	42 389 (1.2)	24 (0.7)	0.10	18 403 (0.7)	74 (1.0)	0.71	2 407 (0.7)	62 (1.3)	5.5
BMI									
<20	78 799 (2.2)	95 (2.9)	0.30	82 829 (3.3)	130 (1.8)	0.47	17 583 (5.3)	74 (1.6)	1.6
20 to <25	557 540 (15.3)	472 (14.5)	0.17	546 156 (21.4)	1005 (13.9)	0.41	95 871 (29.1)	548 (11.9)	1.6
25 to <30	1 264 483 (34.8)	1091 (33.4)	0.16	990 174 (38.9)	2455 (33.8)	0.48	124 122 (37.7)	1501 (32.5)	2.9
30 to <35	1 020 824 (28.1)	906 (27.7)	0.17	598 426 (23.5)	2164 (29.8)	0.69	60 635 (18.4)	1434 (31.0)	5.5
35 to <40	463 931 (12.8)	472 (14.5)	0.19	224 781 (8.8)	986 (13.6)	0.85	21 265 (6.5)	717 (15.5)	7.9
≥40	247 318 (6.8)	230 (7.0)	0.18	104 804 (4.1)	513 (7.1)	0.97	9747 (3.0)	349 (7.5)	8.6

Abbreviations: AUD, alcohol use disorder; BMI, body mass index (calculated as weight in kilograms divided by height in meters squared; ≥30 indicates obesity and ≥25 indicates overweight); FIB-4, Fibrosis-4 Index; HCC, hepatocellular carcinoma.

^a^ Race and ethnicity were self-reported and obtained from the US Department of Veterans Affairs electronic health record.

^b^ Other includes American Indian or Alaska Native, Asian, Native Hawaiian or Other Pacific Islander, and multiracial.

^c^ Alcohol use was classified as history of AUD or, in the absence of AUD, was assessed with Alcohol Use Disorders Identification Test–Consumption and categorized as abstinent (score of 0), low risk (score of 1-2 [females] or 1-3 [males]), moderate risk (score of 3-7 [females] or 4-7 [males]), or high risk (score of ≥8 [females and males]).

In multivariable analysis, in the development sample (model 4 in eTable 1 in Supplement 1), FIB-4 was by far the most important factor, with an overall χ^2^ of 14 967, followed by diabetes status (χ^2^ = 2010) and age (χ^2^ = 899). Discrimination of model 4, as measured by C statistic, was 0.82 (95% CI, 0.81-0.84). The association with age was relatively flat between ages 60 and 79 years, decreased modestly at age 80 years or older, and decreased sharply at age 60 years or younger. Within the FIB-4 strata, there were gradients of risk—for example, among those with FIB-4 higher than 3.25, the hazard ratios (HRs) ranged from 11.7 (95% CI, 10.4-13.1) to 70.5 (95% CI, 62.5-79.4).

When we compared the multivariable model using ordinal values to a model using continuous values and a decomposition of FIB-4 into its component variables, the model’s discrimination improved to 0.83 (95% CI, 0.82-0.85). The best model usng FIB-4 alone had a lower discrimination of 0.79 (95% CI, 0.77-0.80). We chose the continuous model as the final risk score model (Table 3; eFigure 2 in Supplement 1). Using the derived risk score as the only factor in Cox proportional hazards regression models, discrimination was 0.84 (95% CI, 0.83-0.84) in the development sample and 0.82 (95% CI, 0.82-0.82) in the validation sample.

**Table 3.** Cox Proportional Hazards Regression Model Fit to Development Sample of Veterans With Hepatocellular Carcinoma Events During a Maximum 10-Year Follow-Up

Characteristic	PE	SE	χ^2^	*P* value	HR (95% CI)
Age, y					
X = (Age – 50)/5^a^	0.782	0.031	627	<.001	2.19 (2.06-2.32)
X^2^^a^	−0.083	0.009	76	<.001	0.92 (0.90-0.94)
X^3^^a^	0.002	0.001	4	.03	1.00 (1.00-1.00)
Sex					
Male	0 [Reference]	NA	NA	NA	1 [Reference]
Female	−0.616	0.087	50	<.001	0.54 (0.46-0.64)
Race and ethnicity^b^					
Black, non-Hispanic	−0.472	0.039	144	<.001	0.62 (0.58-0.67)
Hispanic	0.566	0.036	242	<.001	1.76 (1.64-1.89)
White, non-Hispanic	0 [Reference]	NA	NA	NA	1 [Reference]
Other^c^	0.049	0.045	1	.28	1.05 (0.96-1.15)
Unknown	0.204	0.036	33	<.001	1.23 (1.14-1.32)
FIB-4 components					
ALT, U/L					
X = (ALT – 30)/10^a^	−0.065	0.012	32	<.001	0.94 (0.92-0.96)
X^2^^a^	0.002	0.004	0	.65	1.00 (0.99-1.01)
X^3^^a^	−0.001	0.000	2	.19	1.00 (1.00-1.00)
AST, U/L					
X = (AST – 30)/10^a^	0.720	0.014	2834	<.001	2.05 (2.00-2.11)
X^2^^a^	−0.067	0.004	241	<.001	0.94 (0.93-0.94)
X^3^^a^	0.002	0.000	30	<.001	1.00 (1.00-1.00)
PLT count per microliter					
X = (PLT – 200)/50^a^	−0.383	0.013	907	<.001	0.68 (0.67-0.70)
X^2^^a^	0.168	0.004	2098	<.001	1.18 (1.17-1.19)
X^3^^a^	−0.011	0.002	48	<.001	0.99 (0.99-0.99)
Diabetes status					
No	0 [Reference]	NA	NA	NA	1 [Reference]
Yes	0.883	0.020	1871	<.001	2.42 (2.32-2.52)
Smoking status					
Never	0 [Reference]	NA	NA	NA	1 [Reference]
Current	0.537	0.027	395	<.001	1.71 (1.62-1.81)
Former	0.171	0.024	50	<.001	1.19 (1.13-1.24)
Alcohol use^d^					
AUD	0.485	0.031	251	<.001	1.62 (1.53-1.72)
Abstinent	0.147	0.024	37	<.001	1.16 (1.11-1.21)
Lower risk	0 [Reference]	NA	NA	NA	1 [Reference]
Moderate risk	0.063	0.040	2	.12	0.94 (0.87-1.02)
High risk	0.202	0.096	4	.03	1.22 (1.02-1.48)
BMI					
X = (BMI – 25)/5^a^	0.165	0.020	66	<.001	1.18 (1.13-1.23)
X^2^^a^	0.023	0.015	2	.13	1.02 (0.99-1.05)
X^3^^a^	−0.008	0.003	8	.005	0.99 (0.99-1.00)

Abbreviations: ALT, alanine aminotransferase; AST, aspartate aminotransferase; AUD, alcohol use disorder; BMI, body mass index (calculated as weight in kilograms divided by height in meters squared; ≥30 indicates obesity and ≥25 indicates overweight); FIB-4, Fibrosis-4 Index; HR, hazard ratio; NA, not applicable; PE, parameter estimate; PLT, platelet.

SI conversion factor: To convert ALT and AST to microkatal per liter, multiply by 0.0167; platelet count to ×10^9^/L, multiply by 1.0.

^a^ X is the tranformation of the variable into its centered value, on a meaningful scale. X^2^ is the square of this value, and X^3^ is the cube. For example, age is centered at 50 years and then divided by 5 so that the HR represents the association of a 5-year increment of age above or below 50 years.

^b^ Race and ethnicity were self-reported and obtained from the US Department of Veterans Affairs electronic health record.

^c^ Other includes American Indian or Alaska Native, Asian, Native Hawaiian or Other Pacific Islander, and multiracial.

^d^ Alcohol use was classified as history of AUD or, in the absence of AUD, was assessed with Alcohol Use Disorders Identification Test–Consumption and categorized as abstinent (score of 0), low risk (score of 1-2 [females] or 1-3 [males]), moderate risk (score of 3-7 [females] or 4-7 [males]), or high risk (score of ≥8 [females and males]).

The HCC risk score we developed was approximately normally distributed, with median (IQR) scores of 41 (33-47) in the development and 43 (35-49) in the validation samples. The HCC risk as a function of score was exponential, meaning that a linear association was observed when plotted with a log_10_ scale on the y-axis (eFigure 3 in Supplement 1). Using first and 99th percentiles of scores among those who experienced HCC within 10 years, incidence of HCC extended across 4 orders of magnitude from 0.4% for a score of 28 to 44.2% for a score of 85. Associations were similar in development and validation samples at 1, 5, and 10 years. Estimated HCC risk vs risk score was similar to that observed in subgroups of females, those with diabetes, AUD, or BMI >35. The CIs for the subgroups largely overlapped the line for the full sample (Figure). This was not true for FIB-4 alone. The final risk score model also discriminated consistently among different age, racial and ethnic groups, and FIB-4 levels (eFigure 4 in Supplement 1).

**Figure.**
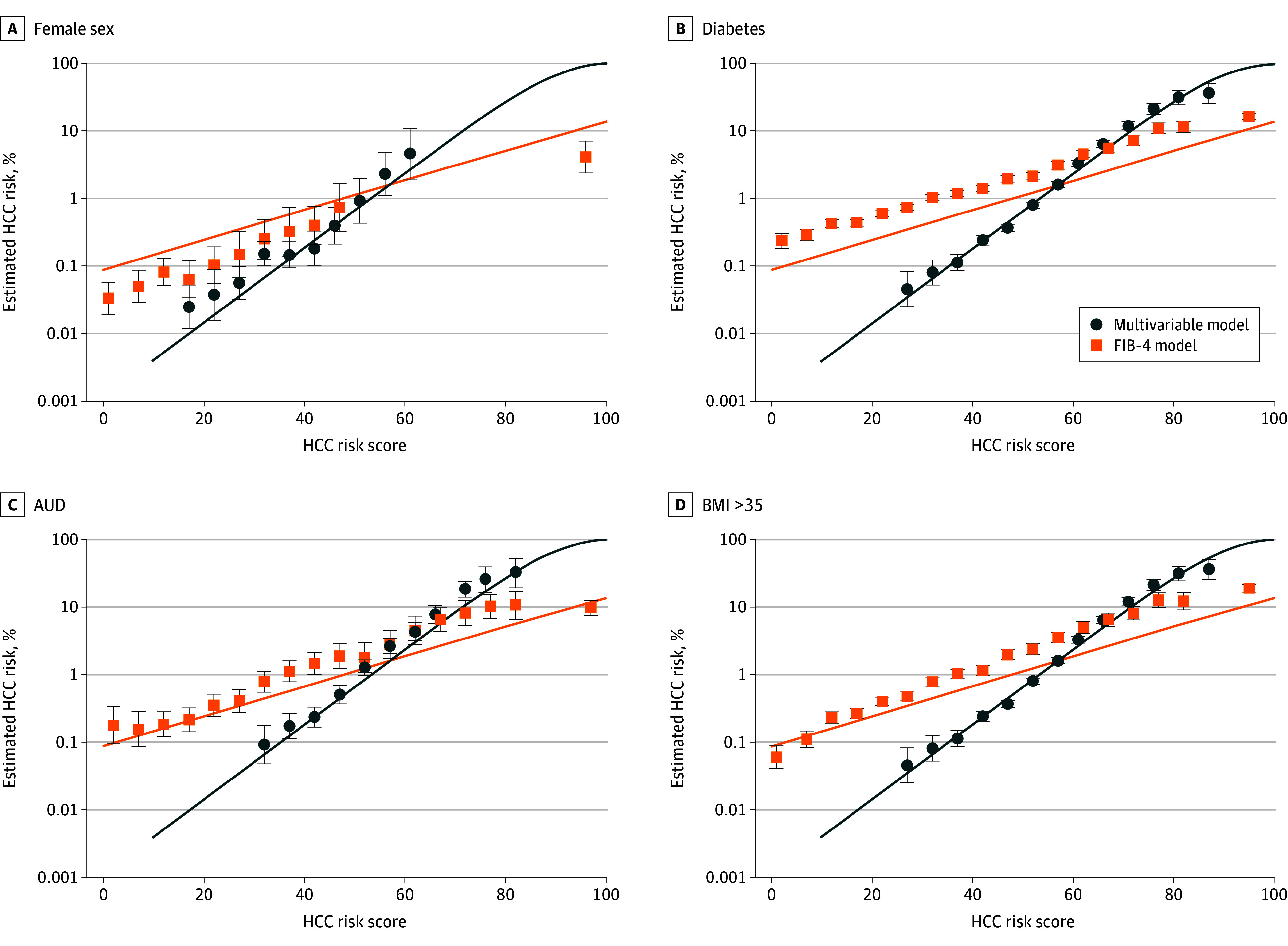
Ten-Year Risk of Hepatocellular Carcinoma (HCC) Lines were estimated using risk scores obtained from the development sample. Subgroup data points from Kaplan-Meier estimates are shown for a minimum of 10 patients with HCC events and 5 who were at risk for HCC at the end of follow-up. Circle represents multivariable model, and square represents Fibrosis-4 Index (FIB-4) model. Error bars represent 95% CIs. AUD indicates alcohol use disorder; BMI, body mass index (calculated as weight in kilograms divided by height in meters squared).

Performance characteristics of this HCC risk score were better than those of FIB-4 alone (Table 4). Using FIB-4 higher than 3.25 as a threshold (5.0% of sample), sensitivity was 38.9% and PPV was 3.5%. For every 29 people screened, 1 true-positive would be detected. Using the model and a score higher than 58 (4.7% of sample), sensitivity was 45.1% and PPV was 4.3%. For every 23 people screened, 1 true-positive would be detected, which represents a 22.9% ([0.043-0.035]/0.035 = 0.229) increase in cancers detected among those who were screened. Of those with risk scores of 58 or higher, 48.6% had FIB-4 under 3.25 (2.8% with a value <1.45), 19.5% were younger than age 65 years, 44.0% did not have diabetes, and 56.7% reported abstinence from alcohol.

**Table 4.** Performance Characteristics of Fibrosis-4 Index Thresholds vs Risk Score Model

Characteristic	**Veterans, No. (%)**	**s(t)**	**1 − s(t)**	**TP, No.**	**FP, No.**	**Sensitivity, %**	**Specificity, %**	**PPV, %**	**No. of FP/No. of TP**
Overall	6 509 288	0.996	0.004	29 292	6 479 996	NA	NA	NA	NA
FIB-4 threshold									
≥1.30	3 398 690 (52.2)	0.993	0.007	25 150	3 373 540	85.9	47.9	0.7	134
≥1.45	2 876 393 (44.2)	0.992	0.008	23 874	2 852 519	81.5	56.0	0.8	119
>2.67	631 461 (9.7)	0.978	0.022	14 082	617 379	48.1	90.5	2.2	44
>3.25	325 531 (5.0)	0.965	0.035	11 394	314 137	38.9	95.2	3.5	28
HCC risk score, ≥^a^									
35	4 711 157 (72.4)	0.994	0.006	28 738	4 682 419	98.1	27.7	0.6	163
40	3 759 250 (57.8)	0.993	0.007	27 443	3 731 807	93.7	42.4	0.7	136
45	2 349 522 (36.1)	0.990	0.010	24 200	2 325 322	82.6	64.1	1.0	96
50	1 182 043 (18.2)	0.983	0.017	19 858	1 162 185	67.8	82.1	1.7	59
51	1 010 783 (15.5)	0.981	0.019	19 003	991 780	64.9	84.7	1.9	52
52	859 608 (13.2)	0.979	0.021	18 138	841 470	61.9	87.0	2.1	46
53	728 425 (11.2)	0.976	0.024	17 191	711 234	58.7	89.0	2.4	41
54	615 507 (9.5)	0.973	0.027	16 434	599 073	56.1	90.8	2.7	36
55	518 447 (8.0)	0.970	0.030	15 553	502 894	53.1	92.2	3.0	32
56	436 343 (6.7)	0.966	0.034	14 748	421 595	50.3	93.5	3.4	29
57	366 620 (5.6)	0.962	0.038	14 005	352 615	47.8	94.6	3.8	25
58	307 518 (4.7)	0.957	0.043	13 223	294 295	45.1	95.5	4.3	22
59	258 260 (4.0)	0.952	0.048	12 448	245 812	42.5	96.2	4.8	20
60	217 027 (3.3)	0.946	0.054	11 763	205 264	40.2	96.8	5.4	17

Abbreviations: FIB-4, Fibrosis-4 Index; FP, false-positive; HCC, hepatocellular carcinoma; NA, not applicable; PPV, positive predictive value; s(t), survival at time t; TP, true-positive.

^a^ The final model includes age, sex, race and ethnicity, FIB-4 components, diabetes status, smoking status, alcohol use, and body mass index (Table 3).

In the sensitivity analysis, we found no substantive differences excluding index visits in 2008 or restricting to AUDIT-C or BMI within 1 year of the index visit. The broader HCC definition increased the number of events in the development sample from 10 896 to 14 430. However, the C statistic decreased from 0.83 (95% CI, 0.82-0.85) to 0.80 (95% CI, 0.77-0.83), and 26 of 41 HRs were attenuated by more than 10% (eTable 2 in Supplement 1).

## Discussion

Screening for HCC could serve both primary and secondary prevention goals if conducted when major risk factors remain modifiable and if it detects HCC at a point when curative treatment remains an option. In this cohort of 6 509 288 veterans without chronic viral hepatitis or decompensated cirrhosis, most incident HCC (69.5%) occurred without advanced hepatic fibrosis (FIB-4 ≤3.25) at baseline. These patients had many modifiable risk factors, including obesity, diabetes, unhealthy alcohol use, and current smoking status, that are commonly associated with cardiovascular disease but less commonly recognized by primary care clinicians as risk factors for HCC.^24,25^ Furthermore, we developed and validated a risk score that identified individuals for whom screening would likely detect HCC at a point when curative treatment may still be an option.

Obesity and diabetes, key players in metabolic syndrome, were independently associated with HCC incidence. The HCC risk associated with obesity is tied to the development of steatotic liver disease, which commonly goes undetected.^26^ Although there have been efforts to update contemporary cohorts for HCC risk in cirrhosis to reflect the increasing prevalence of MASLD-associated cirrhosis,^1^ up to 38.5% of steatotic liver disease–associated HCC emerges in the absence of cirrhosis.^8,9^ The burden of steatotic liver disease and its sequelae (liver cancer and liver failure) continues to increase with the growing obesity epidemic.^2^

The findings of this study suggest that early interventions aimed at weight and glucose control may play a role in effective reduction of HCC risk if implemented before progression to advanced fibrosis. With a better understanding of risk, clinicians can prioritize their approach to treatment and prevention of metabolic dysfunction–associated comorbidities and subsequent liver disease.^27^ Clinicians can also provide patients with a more personalized explanation of HCC risk in the context of patients’ unique comorbidities and lifestyles. This understanding may prompt consideration of interventions aimed at weight loss and glycemic control and may even inform an individualized approach to HCC screening.

Consistent with prior research attributing 30% of all HCC cases to ALD, we found that unhealthy alcohol use and smoking are HCC risk factors in the present cohort. Smoking and alcohol consumption are common companion behaviors, and alcohol, smoking, and obesity are all believed to lead to hepatic injury through chronic inflammation and cellular stress.^28,29^ In this study, the implications of unhealthy alcohol use, whether dose-dependent according to AUDIT-C score or AUD diagnosis, were greater for patients without advanced fibrosis, particularly those with FIB-4 of 1.45 to 3.25. Independent of alcohol use, current smoking status was associated with an increased HCC risk compared with never smoking status, with the greatest risk among patients with FIB-4 higher than 3.25. Smoking is cytotoxic and associated with increased inflammation and fibrosis in the liver through toxic (ie, iron overload and purine catabolism), immunological (ie, proinflammatory cytokines interleukin 1, interleukin 6, and tumor necrosis factor), and oncogenic (ie, p53 and T-cell suppression) properties.^28^ Thus, the potential role of smoking cessation in HCC risk reduction in patients with low FIB-4 scores should not be underestimated.

Not all HCC risk factors are modifiable via health care interventions. Patients in our cohort who identified as Hispanic had the greatest risk for developing HCC, particularly those with FIB-4 higher than 3.25. This phenomenon has been associated with factors ranging from genetics (eg, sequence variation in the *PNPLA3* gene) to sociocultural barriers (eg, lack of access to health care and education) affecting diabetes-related chronic disease in the Hispanic population.^30,31^ These findings highlight the complex intersection of biological and social determinants of health and the importance of raising awareness about liver disease and identifying methods for earlier detection of liver disease and liver cancer in high-risk patients, such as those of Hispanic or Latino ethnicity.

To our knowledge, this study was the first modern large-scale research on the risk factors of HCC in adults without chronic viral hepatitis or hepatic decompensation that accounts for level of liver fibrosis. However, we studied a population of predominantly middle-aged, White, male veterans, with a lower proportion of female veterans (7.1%). The HCC risk score we developed was not meant to flag any one factor as most important but to demonstrate the cumulative outcome of multiple factors and the associated increase in HCC risk. This finding was underscored by the observation that, among individuals with a risk score of 58, which we suggest as a potential threshold for screening, 48.6% had FIB-4 lower than 3.25 and 2.8% had FIB-4 lower than 1.45.

### Limitations

This study has several limitations. The final risk score model has been validated in independent data within the VA health care system and is ready for clinical application in this system. However, the model requires external validation prior to application in other settings, particularly settings in which factors that were not included in the model may play important roles in HCC. Because the study excluded patients with prevalent HCC, associations with age should be interpreted with care. Among patients in the oldest age groups at baseline, most of the HCC events that were going to occur had already occurred. The observational design of this study limited our ability to identify confounding variables not routinely collected in the EHR, including past alcohol consumption among current abstainers. Additionally, rare causes of liver disease (ie, hemochromatosis and autoimmune hepatitis) were included. We did not separately consider steatotic liver disease because we included patient observations from as early as 2007, when MASLD was less recognized and underdiagnosed. Furthermore, there are intrinsic limitations to noninvasive scoring systems, as an individual’s FIB-4 can fluctuate over time and increases with age.^32^ However, studies have validated the diagnostic accuracy of FIB-4, and higher FIB-4 was associated with increased HCC risk even in the absence of a prior diagnosis of cirrhosis.^11^ Additionally, FIB-4 may overestimate the degree of fibrosis in patients with ALD due to elevated liver transaminases.^33^

## Conclusions

Liver disease is often missed in the primary care setting.^26^ Greater attention to obesity, diabetes, unhealthy alcohol use, and smoking in individuals with intermediate FIB-4 values and those of Hispanic ethnicity might better identify people at substantial risk of HCC. In this cohort study of over 6 million adults without viral hepatitis, most HCC cases occurred among patients without advanced fibrosis or cirrhosis, a population for whom HCC screening is not advised. As the incidence of HCC continues to increase in tandem with the burden of obesity, the use of algorithms to stratify patients by HCC risk can guide patient-centered care. We developed an HCC risk score based on widely available clinical variables to provide a personalized approach to HCC risk assessment and mitigation. This risk score outperformed FIB-4 in identifying patients at risk of HCC. Although this risk score has been validated in independent data, future work should include validation of this tool outside of the VA health care system.

## Notes

Supplement 1. **eFigure 1.** Patient Flow Chart **eTable 1.** Cox Models (1-5) Fit to Development Sample of 5 119 775 Veterans With 10,896 Hepatocellular Carcinoma Events in a Maximum of 10 Years Follow-Up **eFigure 2.** Comparison of Cox Models (1-6) Fit to Development Sample of 5,119,775 Veterans With 10 896 Hepatocellular Carcinoma Events in a Maximum of 10 Years Follow-Up **eFigure 3.** Risk of Hepatocellular Carcinoma (HCC), As a Function of HCC Risk Score **eFigure 4.** 10-Year Risk of HCC **eTable 2.** Sensitivity Analysis Cox Model (4) Fit to Subsets of Development Sample With Maximum of 10 Years Follow-Up **eTable 3.** ICD-9 and 10 Codes Used to Define Exclusions and Covariate Conditions

Supplement 2.
Data Sharing Statement
