# Errors in the Results, Tables, and Supplement

**DOI:** 10.1001/jamanetworkopen.2025.50105

**Published:** 2025-12-16

In the Original Investigation, “Risk Score for Hepatocellular Cancer in Adults Without Viral Hepatitis or Cirrhosis,”^1^ published on November 6, 2024, in *JAMA Network Open*, a discrepancy in 1 of the variables used to measure patterns of alcohol use was discovered by the authors after publication. The alcohol use disorder (AUD) variable used did not include the complete history. Correcting this by using diagnosis of AUD ever before the index date resulted in the hazard ratio (HR) for AUD increasing from 1.17 (95% CI, 1.05-1.29) to 1.62 (95% CI, 1.53-1.72). The associations of some related exposures also changed with this correction. The HR for high-risk alcohol use decreased from 1.33 (95% CI, 1.19-1.49) to 1.22 (95% CI, 1.02-1.48), and that for current smoking from 1.81 (95% CI, 1.72-1.91) to 1.71 (95% CI, 1.62-1.72). The alcohol sections in the text, Table 1, and Table 2, all of Table 3, and models 4 and 5 in Supplement 1 are affected. The overall findings, interpretations, and conclusions remain unchanged. The authors have explained what happened in a Comment^2^ posted with the article, and the article has been corrected.

## References

[1] Ilagan-Ying YC, Gordon KS, Tate JP, . Risk score for hepatocellular cancer in adults without viral hepatitis or cirrhosis. JAMA Netw Open. 2024;7(11):e2443608. doi:10.1001/jamanetworkopen.2024.43608 39504020 PMC11541635

[2] Tate JP. Correction. JAMA Netw Open. November 11,2025. Accessed November 12, 2025. https://jamanetwork.com/journals/jamanetworkopen/fullarticle/2825810

