# Supplementary material for: Risk Score for Hepatocellular Cancer in Adults Without Viral Hepatitis or Cirrhosis
Source: JAMA Netw Open. 2024 Nov 6;7(11):e2443608. doi: 10.1001/jamanetworkopen.2024.43608 (PMC11541635; doi:10.1001/jamanetworkopen.2024.43608)
Supplement: Supplement 1. — eFigure 1. Patient Flow Chart eTable 1. Cox Models (1-5) Fit to Development Sample of 5 119 775 Veterans With 10,896 Hepatocellular Carcinoma Events in a Maximum of 10 Years Follow-Up eFigure 2. Comparison of Cox Models (1-6) Fit to Development Sample of 5,119,775 Veterans With 10 896 Hepatocellular Carcinoma Events in a Maximum of 10 Years Follow-Up eFigure 3. Risk of Hepatocellular Carcinoma (HCC), As a Function of HCC Risk Score eFigure 4. 10-Year Risk of HCC eTable 2. Sensitivity Analysis Cox Model (4) Fit to Subsets of Development Sample With Maximum of 10 Years Follow-Up eTable 3. ICD-9 and 10 Codes Used to Define Exclusions and Covariate Conditions [file jamanetwopen-e2443608-s001.pdf]

Supplemental Online Content

Ilagan-Ying YC, Gordon KS, Tate JP, et al. Risk score for hepatocellular cancer in adults without viral hepatitis or cirrhosis. *JAMA Netw Open*. 2024;7(11): e2443608. doi:10.1001/jamanetworkopen.2024.43608

eFigure 1. Patient Flow Chart

eTable 1. Cox Models (1-5) Fit to Development Sample of 5,119,775 Veterans With 10,896 Hepatocellular Carcinoma Events in a Maximum of 10 Years Follow-Up

eFigure 2. Comparison of Cox Models (1-6) Fit to Development Sample of 5,119,775 Veterans With 10,896 Hepatocellular Carcinoma Events in a Maximum of 10 Years Follow-Up

eFigure 3. Risk of Hepatocellular Carcinoma (HCC), As a Function of HCC Risk Score

eFigure 4. 10-Year Risk of HCC

eTable 2. Sensitivity Analysis Cox Model (4) Fit to Subsets of Development Sample With Maximum of 10 Years Follow-Up

eTable 3. ICD-9 and 10 Codes Used to Define Exclusions and Covariate Conditions

This supplemental material has been provided by the authors to give readers additional information about their work.

© 2024 Ilagan-Ying YC et al. *JAMA Network Open*

eFigure 1. Patient flow chart

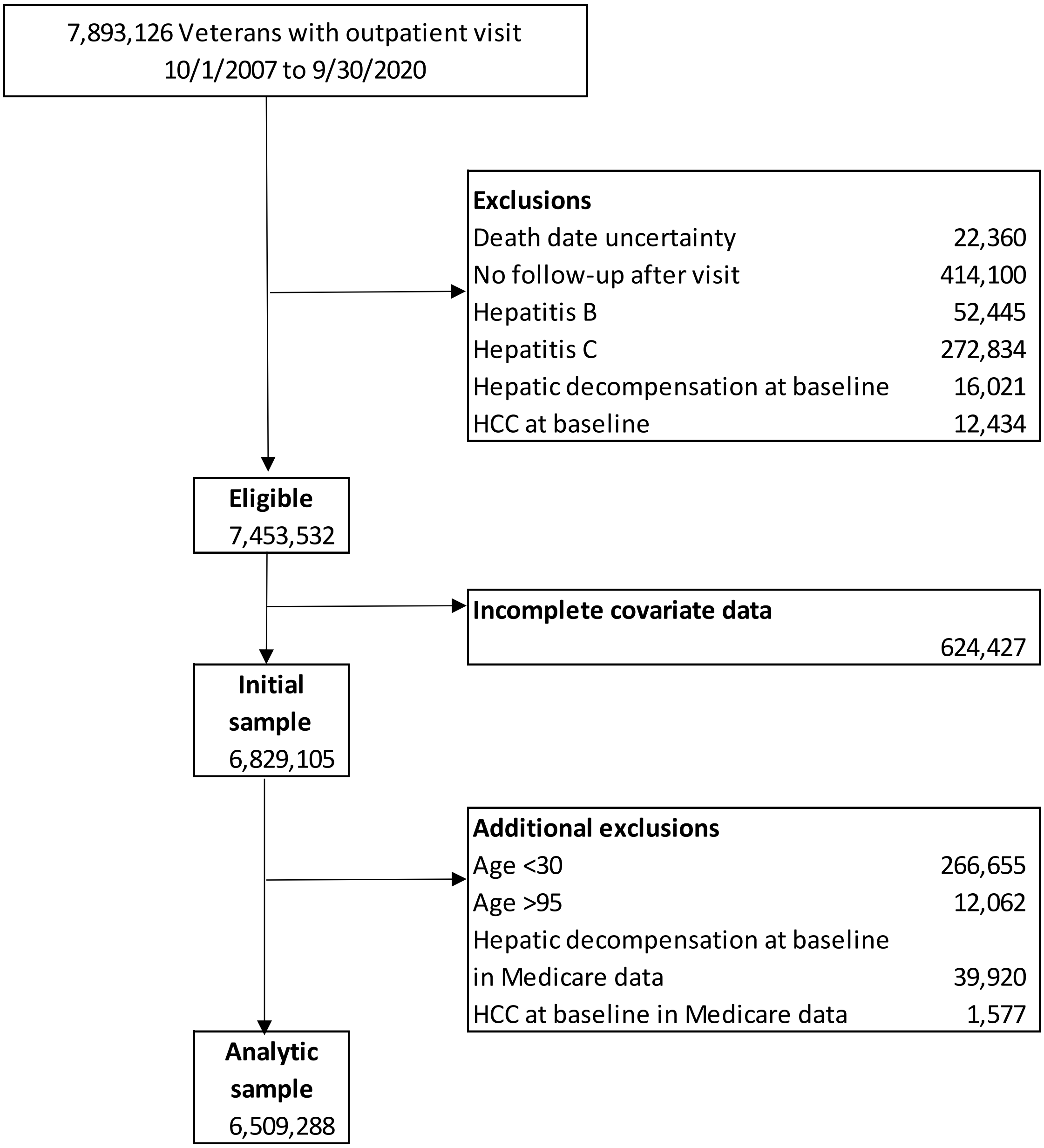

© 2024 Ilagan-Ying YC et al. *JAMA Network Open*

eTable 1. Cox models (1-5) fit to development sample of 5,119,775 veterans with 10,896 hepatocellular carcinoma events in a maximum of 10 years follow-up

Model	Predictor	χ^2 overall	Level or form	Events	PE	SE	χ^2	p	HR	(95% CI)	Risk Score Points
1	FIB-4	13,645	<1.45	2,308							0
			1.45-3.25	5,279	1.247	0.025	2	<.0001	3.48	(3.31, 3.66)	40
			>3.25	3,309	3.072	0.027	12,798	<.0001	21.6	(20.5, 22.8)	100
2	FIB-4	17,926	<.9	520							0
			0.9 to <1.1	552	0.707	0.061	134	<.0001	2.03	(1.80, 2.29)	14
			1.1 to <1.3	671	0.940	0.058	259	<.0001	2.56	(2.28, 2.87)	19
			1.3 to <1.45	565	1.161	0.061	365	<.0001	3.19	(2.83, 3.60)	23
			1.45 to < 1.6	597	1.346	0.060	504	<.0001	3.84	(3.42, 4.32)	27
			1.6 to < 1.8	794	1.528	0.056	734	<.0001	4.61	(4.13, 5.15)	31
			1.8 to <2.0	759	1.724	0.057	917	<.0001	5.61	(5.02, 6.27)	35
			2.0 to < 2.2	715	1.933	0.058	1,124	<.0001	6.91	(6.17, 7.73)	39
			2.2 to <2.4	620	2.068	0.059	1,209	<.0001	7.91	(7.04, 8.88)	42
			2.4 to <2.6	539	2.219	0.061	1,304	<.0001	9.20	(8.16, 10.4)	45
			2.6 to < 3.0	819	2.371	0.056	1,787	<.0001	10.71	(9.59, 11.9)	48
			3.0 to <3.25	456	2.714	0.064	1,790	<.0001	15.09	(13.3, 17.1)	55
			3.26 to < 4.0	956	3.073	0.055	3,181	<.0001	21.6	(19.4, 24.1)	62
			4.0 to < 5.0	832	3.692	0.056	4,360	<.0001	40.1	(36.0, 44.8)	74
			5.0 to < 6.0	498	4.198	0.063	4,480	<.0001	66.5	(58.8, 75.2)	85
			6.0 to <7.0	311	4.543	0.072	4,013	<.0001	94.0	(81.6, 108)	92
			≥ 7.0	692	4.964	0.058	7,302	<.0001	143.2	(128, 160)	100
3	FIB-4	2,762	X= (FIB-4 - 1.33)		1.207	0.023	2,762	<.0001	3.34	(3.20, 3.50)	
		172	X^2		-0.145	0.011	172	<.0001	0.87	(0.85, 0.88)	
		41	X^3		0.008	0.001	41	<.0001	1.01	(1.01, 1.01)	

© 2024 Ilagan-Ying YC et al. *JAMA Network Open*

4	Age	885	<45	82	-2.05	0.12	308	<.0001	0.13	(0.10, 0.16)	-19
			45-49	109	-1.28	0.10	163	<.0001	0.28	(0.23, 0.34)	-12
			50-54	251	-0.86	0.07	158	<.0001	0.43	(0.37, 0.49)	-8
			55-59	709	-0.30	0.04	44	<.0001	0.74	(0.68, 0.81)	-3
			60-64	1,832							0
			65-69	2,316	0.09	0.03	9	0.0034	1.10	(1.03, 1.17)	1
			70-74	1,988	0.10	0.03	9	0.0034	1.10	(1.03, 1.18)	1
			75-79	1,730	0.07	0.04	3	0.0651	1.07	(1.00, 1.15)	1
			≥80	1,879	-0.36	0.04	95	<.0001	0.70	(0.65, 0.75)	-3
	Sex	60	Male	10,760							0
			Female	136	-0.67	0.09	60	<.0001	0.51	(0.43, 0.60)	-6
	Race	520	White	7,898							0
			Black	734	-0.56	0.04	203	<.0001	0.57	(0.53, 0.62)	-5
			Hispanic	855	0.57	0.04	243	<.0001	1.76	(1.64, 1.89)	5
			Other	519	0.03	0.05	0	0.523	1.03	(0.94, 1.13)	0
			Unknown	890	0.18	0.04	25	<.0001	1.20	(1.12, 1.28)	2
	FIB4	14,521	<.9	520							0
			0.9 to <1.1	552	0.18	0.06	8	0.0037	1.20	(1.06, 1.35)	2
			1.1 to <1.3	671	0.27	0.06	20	<.0001	1.31	(1.16, 1.47)	2
			1.3 to <1.45	565	0.43	0.06	46	<.0001	1.53	(1.35, 1.73)	4
			1.45 to < 1.6	597	0.59	0.06	88	<.0001	1.80	(1.59, 2.03)	5
			1.6 to < 1.8	794	0.76	0.06	166	<.0001	2.15	(1.91, 2.41)	7
			1.8 to <2.0	759	0.97	0.06	264	<.0001	2.65	(2.35, 2.98)	9
			2.0 to < 2.2	715	1.20	0.06	391	<.0001	3.33	(2.95, 3.75)	11
			2.2 to <2.4	620	1.35	0.06	467	<.0001	3.88	(3.43, 4.38)	13
			2.4 to <2.6	539	1.52	0.06	555	<.0001	4.60	(4.05, 5.22)	14
			2.6 to < 3.0	819	1.71	0.06	817	<.0001	5.52	(4.91, 6.21)	16
			3.0 to <3.25	456	2.07	0.07	940	<.0001	7.92	(6.94, 9.03)	19
			3.26 to < 4.0	956	2.43	0.06	1,731	<.0001	11.3	(10.1, 12.7)	23
			4.0 to < 5.0	832	3.02	0.06	2,574	<.0001	20.5	(18.2, 23.0)	28
			5.0 to < 6.0	498	3.48	0.07	2,801	<.0001	32.4	(28.5, 36.8)	33
			6.0 to <7.0	311	3.81	0.07	2,638	<.0001	45.3	(39.1, 52.4)	36
			≥ 7.0	692	4.18	0.06	4,702	<.0001	65.6	(58.2, 74.0)	39
	Diabetes	2,024	No	6,203							0
			Yes	4,693	0.92	0.02	2,024	<.0001	2.50	(2.40, 2.60)	9
	Smoking	369	Never	2,809							0
			Current	3,445	0.51	0.03	359	<.0001	1.67	(1.58, 1.76)	5
			Former	4,642	0.19	0.02	62	<.0001	1.21	(1.15, 1.27)	2
	Alcohol	395	AUD	5,038	0.56	0.03	341	<.0001	1.75	(1.65, 1.86)	5
			Abstinent	2,714	0.12	0.02	25	<.0001	1.13	(1.08, 1.18)	1
			Lower risk	808							0
			Moderate risk	115	-0.02	0.04	0	0.7032	0.99	(0.91, 1.07)	0
			High risk	2,221	0.32	0.10	11	0.0008	1.38	(1.14, 1.66)	3
	BMI	454	<20	219	0.06	0.07	1	0.3989	1.06	(0.92, 1.23)	1
			20 to <25	1,472							0
			25 to <30	3,702	0.25	0.03	62	<.0001	1.28	(1.20, 1.36)	2
			30 to <35	3,210	0.50	0.03	237	<.0001	1.65	(1.55, 1.76)	5
			35 to <40	1,524	0.65	0.04	294	<.0001	1.92	(1.78, 2.07)	6
			>= 40	769	0.68	0.05	212	<.0001	1.97	(1.80, 2.15)	6

© 2024 Ilagan-Ying YC et al. *JAMA Network Open*

Age	374	X= (Age - 50)/5		0.622	0.032	374	<.0001	1.86	(1.75, 1.98)
	90	X^2		-0.091	0.010	90	<.0001	0.91	(0.90, 0.93)
	7	X^3		0.002	0.001	7	0.0104	1.00	(1.00, 1.00)
Sex	55	Male	10,760						
		Female	136	-0.647	0.087	55	<.0001	0.52	(0.44, 0.62)
Race	516	White	7,898						(0.00, 0.00)
		Black	734	-0.548	0.039	196	<.0001	0.58	(0.54, 0.62)
		Hispanic	855	0.570	0.036	245	<.0001	1.77	(1.65, 1.90)
		Other	519	0.033	0.045	1	0.46	1.03	(0.95, 1.13)
		Unknown	890	0.183	0.036	27	<.0001	1.20	(1.12, 1.29)
FIB4	1276	X= (FIB-4 - 1.33)/10		0.966	0.027	1276	<.0001	2.63	(2.49, 2.77)
	11	X^2		-0.040	0.012	11	0.0009	0.96	(0.94, 0.98)
	5	X^3		-0.003	0.001	5	0.0242	1.00	(0.99, 1.00)
Diabetes	1971	No	6,203						
		Yes	4,693	0.904	0.020	1971	<.0001	2.47	(2.37, 2.57)
Smoking	373	Never	2,809						
		Current	3,445	0.513	0.027	362	<.0001	1.67	(1.59, 1.76)
		Former	4,642	0.185	0.024	58	<.0001	1.20	(1.15, 1.26)
Alcohol	391	AUD	5,038	0.558	0.030	340	<.0001	1.75	(1.65, 1.85)
		Abstinent	2,714	0.125	0.024	27	<.0001	1.13	
		Lower risk	808						(0.00, 0.00)
		Moderate risk	115	-0.013	0.040	0	0.740	0.99	(0.91, 1.07)
		High risk	2,221	0.320	0.096	11	0.0008	1.38	(1.14, 1.66)
BMI	116	X= (BMI - 25)/5		0.217	0.020	116	<.0001	1.24	(1.19, 1.29)
	5	X^2		0.035	0.015	5	0.022	1.04	(1.01, 1.07)
	18	X^3		-0.013	0.003	18	<.0001	0.99	(0.98, 0.99)

© 2024 Ilagan-Ying YC et al. *JAMA Network Open*

eFigure 2. Comparison of Cox models (1-6) fit to development sample of 5,119,775 veterans with 10,896 hepatocellular carcinoma events in a maximum of 10 years follow-up

Model	DF	AIC	c-statistic (95% CI)
1 FIB-4 alone, 3 categories	2	313,150	0.745 (0.729, 0.761)
2 FIB-4 alone, 17 categories	16	310,406	0.783 (0.767, 0.799)
3 FIB-4 alone, continuous	2	310,297	0.785 (0.769, 0.801)
4 Full model, categorical	41	304,630	0.822 (0.808, 0.836)
5 Full model, continuous	21	304,399	0.821 (0.807, 0.835)
6 Full model, continuous, FIB-4 components	27	301,992	0.834 (0.820, 0.847)
Using score			
Development sample	1		0.835 (0.827, 0.835)
Validation sample	1		0.823 (0.816, 0.823)

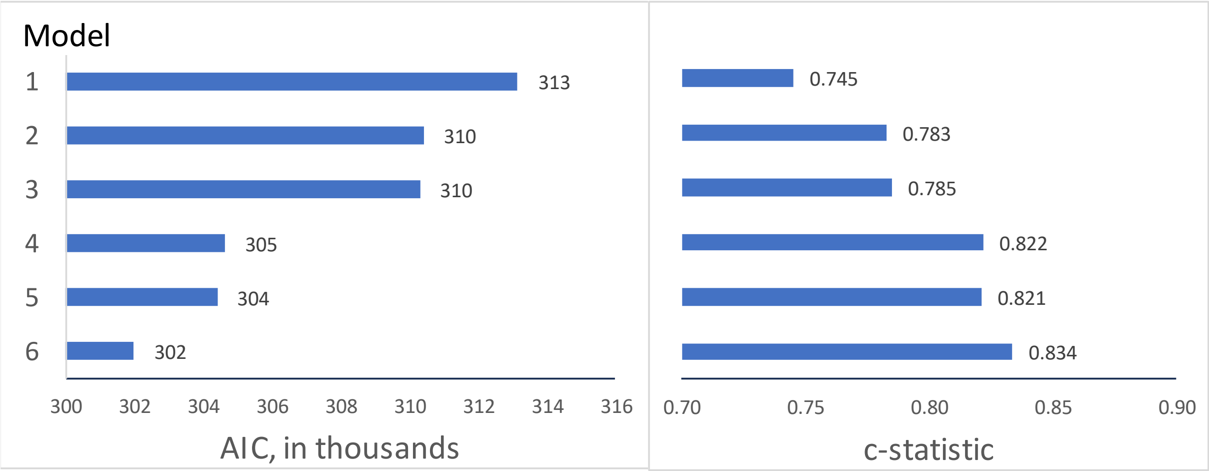

1 FIB-4, 3 categories (<1.45, 1.45-3.25, >3.25)
2 FIB-4, 17 categories
3 FIB-4, continuous
4 Multivariable* model, categorical
5 Multivariable model, continuous
6 Multivariable model, continuous, FIB-4 components

* Age, sex, race, FIB-4, diabetes, smoking, alcohol, BMI

© 2024 Ilagan-Ying YC et al. *JAMA Network Open*

eFigure 3. Risk of hepatocellular carcinoma (HCC), as a function of HCC risk score

Lines are predicted using risk score obtained from development sample. Individual data points from Kaplan-Meier estimates, shown for minimum 10 HCC events and 5 remaining at risk at end of follow-up.

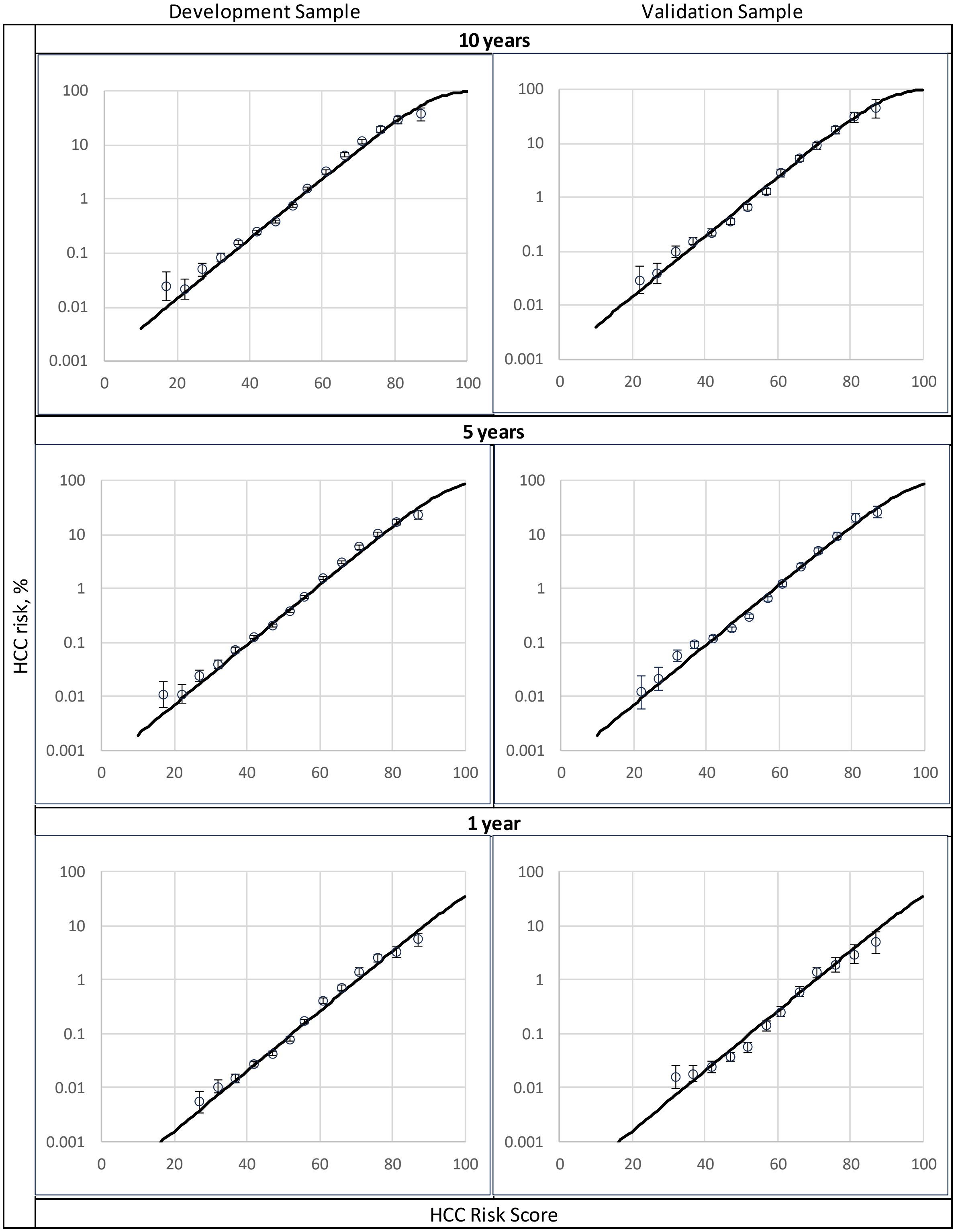

© 2024 Ilagan-Ying YC et al. *JAMA Network Open*

eFigure 4. 10-year risk of HCC

Lines are predicted using risk score obtained from development sample. Black: Multivariable model (age, sex, race, FIB-4 components, diabetes, smoking, alcohol, BMI); Gray: FIB-4, continuous. Subgroups overlaid on full sample. Subgroup data points from Kaplan-Meier estimates, shown for minimum 10 HCC events and 5 remaining at risk at end of follow-up. Circle: Multivariable model, Square: FIB-4 model.

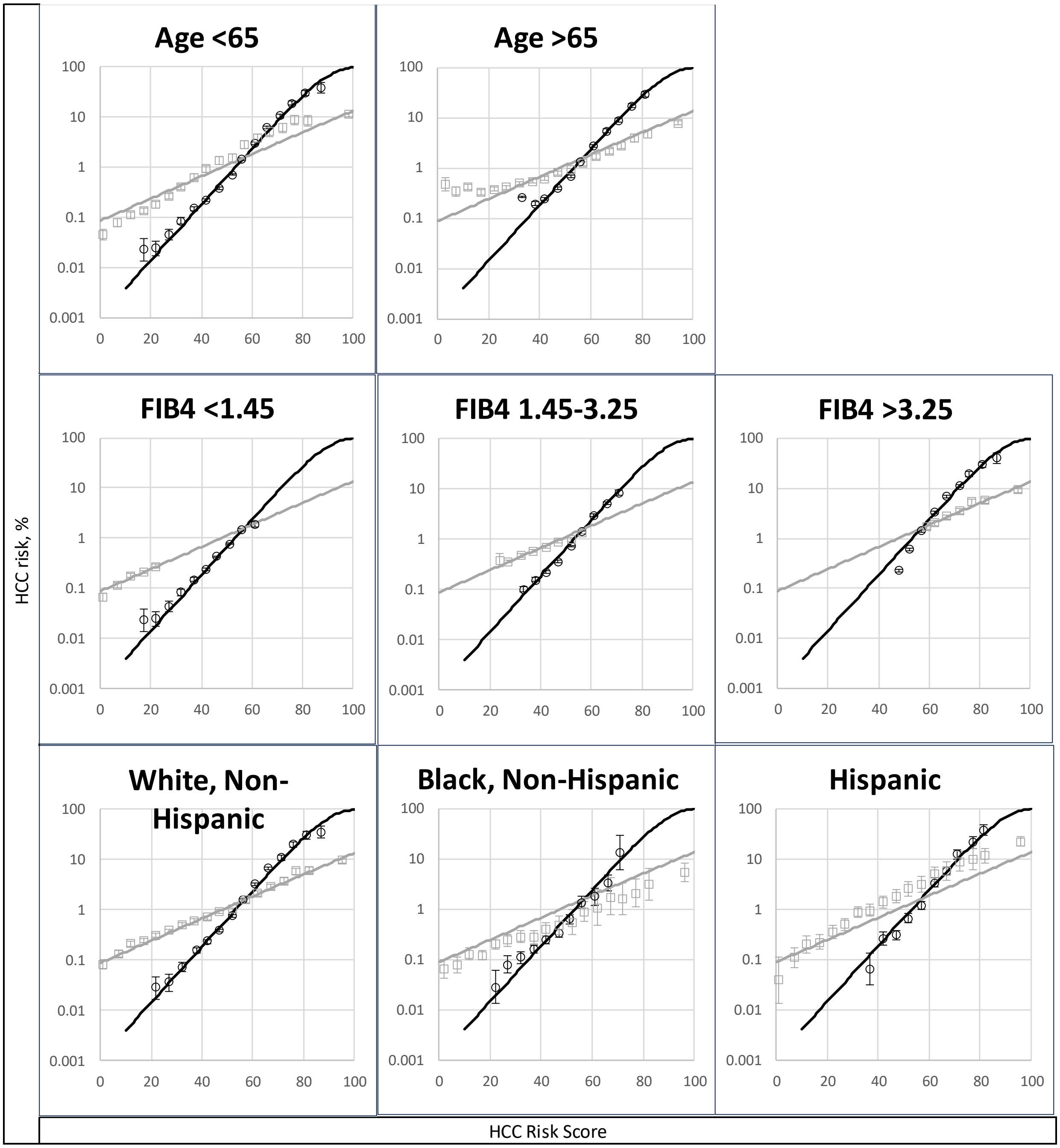

© 2024 Ilagan-Ying YC et al. *JAMA Network Open*

eTable 2. Sensitivity Analysis Cox model (4) fit to subsets of development sample with maximum of 10 years follow-up

		Exclude visits in 2008			Restrict to AUDIT-C within one year			Restrict to BMI within one year			Expand outcome to include ICD codes 155.1, C22.7 and C22.9 (exclude 990 additional present at baseline)		
N		4,888,928			4,995,238			3,648,096			5,119,117		
HCC events		9,960			10,691			8,276			14,430		
c-statistic		0.829 (0.797, 0.861)			0.830 (0.800, 0.861)			0.827 (0.798, 0.855)			0.798 (0.769,0.826)		
		χ^2	HR	(95% CI)	χ^2	HR	(95% CI)	χ^2	HR	(95% CI)	χ^2	HR	(95% CI)
Age	<45	288	0.13	(0.10, 0.16)	298	0.13	(0.10, 0.16)	244	0.13	(0.10, 0.16)	542	0.11	(0.10, 0.14)
	45-49	147	0.28	(0.23, 0.35)	157	0.28	(0.23, 0.34)	126	0.29	(0.23, 0.36)	253	0.28	(0.24, 0.33)
	50-54	143	0.42	(0.37, 0.49)	152	0.43	(0.37, 0.49)	113	0.45	(0.39, 0.52)	203	0.46	(0.41, 0.51)
	55-59	40	0.74	(0.68, 0.81)	39	0.75	(0.69, 0.82)	30	0.76	(0.69, 0.84)	57	0.75	(0.70, 0.81)
	60-64		1.00			1.00			1.00			1.00	
	65-69	7	1.09	(1.02, 1.16)	8	1.09	(1.03, 1.16)	4	1.07	(1.00, 1.15)	14	1.11	(1.05, 1.17)
	70-74	6	1.09	(1.02, 1.17)	6	1.09	(1.02, 1.16)	2	1.05	(0.98, 1.13)	13	1.11	(1.05, 1.18)
	75-79	1	1.04	(0.97, 1.12)	0	1.02	(0.95, 1.10)	0	0.98	(0.90, 1.06)	10	1.11	(1.04, 1.18)
	≥80	117	0.66	(0.61, 0.71)	124	0.66	(0.61, 0.71)	115	0.64	(0.59, 0.70)	59	0.78	(0.73, 0.83)
Sex	Male		1.00			1.00			1.00			1.00	
	Female	64	0.48	(0.40, 0.57)	65	0.49	(0.41, 0.58)	46	0.50	(0.41, 0.61)	59	0.61	(0.53, 0.69)
Race	White		1.00			1.00			1.00			1.00	
	Black	163	0.60	(0.55, 0.65)	163	0.60	(0.56, 0.65)	132	0.59	(0.54, 0.65)	137	0.70	(0.66, 0.74)
	Hispanic	245	1.81	(1.68, 1.95)	257	1.81	(1.68, 1.94)	207	1.83	(1.68, 1.98)	157	1.53	(1.43, 1.64)
	Other	1	1.05	(0.96, 1.15)	2	1.06	(0.97, 1.16)	0	1.03	(0.93, 1.15)	0	1.01	(0.94, 1.09)
	Unknown	14	1.15	(1.07, 1.25)	21	1.18	(1.10, 1.26)	27	1.23	(1.14, 1.32)	49	1.25	(1.17, 1.33)
FIB4	<.9		1.00			1.00			1.00			1.00	
	0.9 to <1.1	8	1.20	(1.06, 1.36)	9	1.21	(1.07, 1.37)	4	1.15	(1.00, 1.33)	3	1.09	(0.99, 1.20)
	1.1 to <1.3	18	1.31	(1.16, 1.48)	18	1.30	(1.15, 1.46)	17	1.32	(1.16, 1.51)	10	1.16	(1.06, 1.27)
	1.3 to <1.45	38	1.50	(1.32, 1.71)	43	1.52	(1.34, 1.72)	39	1.56	(1.36, 1.80)	32	1.31	(1.20, 1.45)
	1.45 to < 1.6	71	1.74	(1.53, 1.98)	90	1.82	(1.61, 2.06)	80	1.88	(1.64, 2.16)	74	1.52	(1.38, 1.67)
	1.6 to < 1.8	146	2.12	(1.87, 2.39)	164	2.16	(1.92, 2.42)	130	2.16	(1.89, 2.47)	137	1.72	(1.57, 1.88)
	1.8 to <2.0	235	2.62	(2.31, 2.96)	260	2.66	(2.36, 2.99)	211	2.70	(2.36, 3.09)	217	2.01	(1.83, 2.21)
	2.0 to < 2.2	367	3.37	(2.98, 3.82)	389	3.36	(2.98, 3.79)	303	3.36	(2.93, 3.85)	323	2.40	(2.18, 2.64)
	2.2 to <2.4	434	3.92	(3.44, 4.45)	469	3.94	(3.48, 4.46)	366	3.95	(3.43, 4.55)	371	2.67	(2.42, 2.95)
	2.4 to <2.6	505	4.59	(4.02, 5.24)	543	4.61	(4.06, 5.24)	473	4.92	(4.26, 5.68)	486	3.20	(2.89, 3.55)
	2.6 to < 3.0	762	5.61	(4.96, 6.34)	821	5.64	(5.01, 6.35)	669	5.81	(5.08, 6.64)	697	3.61	(3.28, 3.97)
	3.0 to <3.25	885	8.13	(7.08, 9.33)	932	8.05	(7.04, 9.20)	733	8.11	(6.97, 9.44)	817	5.06	(4.53, 5.66)
	3.26 to < 4.0	1,602	11.5	(10.2, 13.0)	1,719	11.6	(10.3, 13.0)	1,359	11.7	(10.3, 13.4)	1,641	7.0	(6.3, 7.6)
	4.0 to < 5.0	2,412	21.2	(18.8, 24.0)	2,576	21.3	(18.9, 24.0)	1,963	20.8	(18.2, 23.8)	2,557	12.3	(11.1, 13.5)
	5.0 to < 6.0	2,682	34.4	(30.1, 39.4)	2,827	34.2	(30.0, 38.9)	2,184	33.8	(29.2, 39.2)	2,707	19.1	(17.1, 21.4)
	6.0 to <7.0	2,473	47.4	(40.7, 55.2)	2,641	47.5	(41.0, 55.0)	2,135	49.4	(41.8, 58.2)	2,394	26.1	(22.9, 29.8)
	≥ 7.0	4,495	71.2	(62.8, 80.6)	4,783	71.0	(62.9, 80.2)	3,530	66.5	(57.9, 76.4)	4,970	38.7	(35.0, 42.9)
Diabetes	No		1.00			1.00			1.00			1.00	
	Yes	1,813	2.49	(2.38, 2.59)	1,990	2.50	(2.41, 2.61)	1,577	2.52	(2.41, 2.64)	2,099	2.28	(2.20, 2.36)
Smoking	Never		1.00			1.00			1.00			1.00	
	Current	442	1.80	(1.70, 1.90)	455	1.78	(1.68, 1.87)	358	1.78	(1.68, 1.89)	760	1.88	(1.80, 1.97)
	Former	67	1.23	(1.17, 1.29)	64	1.22	(1.16, 1.27)	46	1.21	(1.14, 1.27)	80	1.21	(1.16, 1.26)
Alcohol	Abstinent	22	1.12	(1.07, 1.17)	19	1.10	(1.06, 1.15)	13	1.10	(1.04, 1.15)	39	1.13	(1.09, 1.17)
	Lower risk		1.00			1.00			1.00			1.00	
	Moderate ri	12	1.13	(1.05, 1.21)	18	1.15	(1.08, 1.23)	22	1.19	(1.11, 1.28)	14	1.11	(1.05, 1.18)
	High risk	53	1.55	(1.38, 1.74)	54	1.53	(1.37, 1.71)	41	1.51	(1.33, 1.71)	75	1.55	(1.40, 1.71)
	AUD	31	1.36	(1.22, 1.51)	27	1.32	(1.19, 1.47)	15	1.29	(1.14, 1.47)	2	1.07	(0.97, 1.17)
BMI	<20	3	1.14	(0.98, 1.32)	1	1.09	(0.94, 1.26)	3	1.16	(0.98, 1.36)	18	1.27	(1.14, 1.41)
	20 to <25		1.00			1.00			1.00			1.00	
	25 to <30	53	1.27	(1.19, 1.36)	57	1.27	(1.19, 1.35)	35	1.23	(1.15, 1.32)	20	1.12	(1.07, 1.18)
	30 to <35	198	1.62	(1.51, 1.73)	224	1.64	(1.53, 1.74)	156	1.59	(1.48, 1.71)	115	1.34	(1.27, 1.41)
	35 to <40	252	1.88	(1.74, 2.04)	276	1.90	(1.76, 2.05)	183	1.81	(1.66, 1.97)	153	1.50	(1.41, 1.60)
	>= 40	193	1.96	(1.78, 2.15)	198	1.93	(1.76, 2.12)	118	1.80	(1.62, 2.00)	104	1.51	(1.40, 1.64)

© 2024 Ilagan-Ying YC et al. *JAMA Network Open*

eTable 3. ICD-9 and 10 codes used to define exclusions and covariate conditions

Condition	Description	ICD-9	ICD-10
Alcohol Use Disorder (AUD)			
	Nondependent alcohol abuse		
	Unspecified	305.00	
	Continuous	305.01	
	Episodic	305.02	
	In remission	305.03	
	Acute alcoholic intoxication		
	Unspecified	303.00	
	Continuous	303.01	
	Episodic	303.02	
	In remission	303.03	
	Other and unspecified alcohol dependence		
	Unspecified	303.90	
	Continuous	303.91	
	Episodic	303.92	
	In remission	303.93	
	Alcohol abuse, uncomplicated		F10.10
	Alcohol abuse with intoxication, uncomplicated		F10.120
	Alcohol abuse with intoxication delirium		F10.121
	Alcohol abuse with intoxication, unspecified		F10.129
	Alcohol abuse with alcohol-induced mood disorder		F10.14
	Alcohol abuse with alcohol-induced psychotic disorder with delusions		F10.150
	Alcohol abuse with alcohol-induced psychotic disorder with hallucinations		F10.151
	Alcohol abuse with alcohol-induced psychotic disorder, unspecified		F10.159
	Alcohol abuse with alcohol-induced anxiety disorder		F10.180
	Alcohol abuse with alcohol-induced sexual dysfunction		F10.181
	Alcohol abuse with alcohol-induced sleep disorder		F10.182
	Alcohol abuse with other alcohol-induced disorder		F10.188
	Alcohol abuse with unspecified alcohol-induced disorder		F10.19
	Alcohol dependence, uncomplicated		F10.20
	Alcohol dependence, in remission		F10.21
	Alcohol dependence with intoxication, uncomplicated		F10.220
	Alcohol dependence with intoxication delirium		F10.221
	Alcohol dependence with intoxication, unspecified		F10.229
	Alcohol dependence with withdrawal, uncomplicated		F10.230
	Alcohol dependence with withdrawal delirium		F10.231
	Alcohol dependence with withdrawal with perceptual disturbance		F10.232
	Alcohol dependence with withdrawal, unspecified		F10.239
	Alcohol dependence with alcohol-induced mood disorder		F10.24

	Alcohol dependence with alcohol-induced psychotic disorder with delusions		F10.250
	Alcohol dependence with alcohol-induced psychotic disorder with hallucinations		F10.251
	Alcohol dependence with alcohol-induced psychotic disorder, unspecified		F10.259
	Alcohol dependence with alcohol-induced persisting amnestic disorder		F10.26
	Alcohol dependence with alcohol-induced persisting dementia		F10.27
	Alcohol dependence with alcohol-induced anxiety disorder		F10.280
	Alcohol dependence with alcohol-induced sexual dysfunction		F10.281
	Alcohol dependence with alcohol-induced sleep disorder		F10.282
	Alcohol dependence with other alcohol-induced disorder		F10.288
	Alcohol dependence with unspecified alcohol-induced disorder		F10.29
Diabetes			
	Diabetes mellitus without mention of complication	250.0x	
	Diabetes with ketoacidosis	250.1x	
	Diabetes with hyperosmolarity	250.2x	
	Diabetes with other coma	250.3x	
	Diabetes with other specified manifestations	250.8x	
	Diabetes with unspecified complication	250.9x	
	Diabetes with renal manifestations	250.4x	
	Diabetes with ophthalmic manifestations	250.5x	
	Diabetes with neurological manifestations	250.6x	
	Diabetes with peripheral circulatory disorders	250.7x	
	Type 1 diabetes mellitus with ketoacidosis		E10.1x
	Type 1 diabetes mellitus with diabetic nephropathy		E10.21
	Type 1 diabetes mellitus with diabetic chronic kidney disease		E10.22
	Type 1 diabetes mellitus with other diabetic kidney complication		E10.29
	Type 1 diabetes mellitus with diabetic retinopathy		E10.3x
	Type 1 diabetes mellitus with diabetic neuropathy		E10.4x
	Type 1 diabetes mellitus with diabetic peripheral angiopathy		E10.5x
	Type 1 diabetes mellitus with diabetic arthropathy		E10.61x
	Type 1 diabetes mellitus with skin complications		E10.62x
	Type 1 diabetes mellitus with oral complications		E10.63x
	Type 1 diabetes mellitus with hypoglycemia		E10.64x
	Type 1 diabetes mellitus with hyperglycemia		E10.65
	Type 1 diabetes mellitus with other specified complication		E10.69
	Type 1 diabetes mellitus with unspecified complications		E10.8
	Type 1 diabetes mellitus without complications		E10.9
	Type 2 diabetes mellitus with hyperosmolarity		E11.0x
	Type 2 diabetes mellitus with ketoacidosis		E11.1x
	Type 2 diabetes mellitus with diabetic nephropathy		E11.21
	Type 2 diabetes mellitus with diabetic chronic kidney disease		E11.22
	Type 2 diabetes mellitus with other diabetic kidney complication		E11.29

© 2024 Ilagan-Ying YC et al. *JAMA Network Open*

	Type 2 diabetes mellitus with diabetic retinopathy		E11.3x
	Type 2 diabetes mellitus with diabetic neuropathy		E11.4x
	Type 2 diabetes mellitus with diabetic peripheral angiopathy		E11.5x
	Type 2 diabetes mellitus with diabetic arthropathy		E11.61x
	Type 2 diabetes mellitus with skin complications		E11.62x
	Type 2 diabetes mellitus with oral complications		E11.63x
	Type 2 diabetes mellitus with hypoglycemia		E11.64x
	Type 2 diabetes mellitus with hyperglycemia		E11.65
	Type 2 diabetes mellitus with other specified complication		E11.69
	Type 2 diabetes mellitus with unspecified complications		E11.8
	Type 2 diabetes mellitus without complications		E11.9
	Other specified diabetes mellitus with hyperosmolarity		E13.0x
	Other specified diabetes mellitus with ketoacidosis		E13.1x
	Other specified diabetes mellitus with diabetic nephropathy		E13.21
	Other specified diabetes mellitus with diabetic chronic kidney disease		E13.22
	Other specified diabetes mellitus with other diabetic kidney complication		E13.29
	Other specified diabetes mellitus with diabetic retinopathy		E13.3x
	Other specified diabetes mellitus with diabetic neuropathy		E13.4x
	Other specified diabetes mellitus with diabetic peripheral angiopathy		E13.5x
	Other specified diabetes mellitus with diabetic arthropathy		E13.61x
	Other specified diabetes mellitus with skin complications		E13.62x
	Other specified diabetes mellitus with oral complications		E13.63x
	Other specified diabetes mellitus with hypoglycemia		E13.64x
	Other specified diabetes mellitus with hyperglycemia		E13.65
	Other specified diabetes mellitus with other specified complication		E13.69
	Other specified diabetes mellitus with unspecified complications		E13.8
	Other specified diabetes mellitus without complications		E13.9
Hepatitis B			
	Chronic viral hepatitis B w/ hepatic coma w/o mention of hepatitis delta	070.22	
	Chronic viral hepatitis B w/ hepatic coma w/ hepatitis delta	070.23	
	Chronic viral hepatitis B w/o mention of hepatic coma w/o mention of hepatitis delta	070.32	
	Chronic viral hepatitis B w/o mention of hepatic coma w/ hepatitis delta	070.33	
	Chronic viral hepatitis B with delta-agent		B18.0
	Chronic viral hepatitis B without delta-agent		B18.1
Hepatitis C			
	Chronic hepatitis C w/ hepatic coma	070.44	
	Chronic hepatitis C w/o mention of hepatic coma	070.54	
	Chronic viral hepatitis C		B18.2

Hepatic decompensation defined by stand-alone condition(s) or ascites with a concurrent liver related disorder

	Description	ICD-9	ICD-10

Stand-alone conditions			
	Esophageal varices with bleeding	456.0	I85.01
	Spontaneous bacterial peritonitis	567.23	K65.2
	Other suppurative peritonitis	567.2	
	Other specified peritonitis	567.8	
	Alcoholic hepatitis with ascites		K70.11
	Alcoholic cirrhosis of liver with ascites		K70.31
	Alcoholic hepatic failure with coma		K70.41
	Hepatic coma, (encephalopathy)	572.2	
	Chronic hepatic failure without coma		K72.10
	Chronic hepatic failure with coma		K72.11
	Hepatic failure, unspecified, with coma		K72.91
	Hepatic encephalopathy		K76.82
Ascites			
	Ascites	789.5	
	Other ascites	789.59	R18.8
Liver related disorders			
	Esophageal varices without bleeding	456.1	I85.00
	Esophageal varices in diseases classified elsewhere, without bleeding	456.21	
	Secondary esophageal varices, without bleeding		I85.10
	Esophageal varices in diseases classified elsewhere, with bleeding	456.20	
	Secondary esophageal varices, with bleeding		I85.11
	Alcoholic cirrhosis of liver	571.2	
	Alcoholic cirrhosis of liver without ascites		K70.30
	Cirrhosis of liver without mention of alcohol	571.5	
	Unspecified cirrhosis of liver		K74.60
	Other cirrhosis of liver		K74.69
	Other chronic nonalcoholic liver disease	571.8	
	Unspecified chronic liver disease w/o mention of alcohol	571.9	
	Other chronic hepatitis	571.49	
	Other sequelae of chronic liver disease	572.8	
	Hepatitis, unspecified	573.3	
	Unspecified disorder of liver	573.9	
	Alcoholic hepatitis, without ascites		K70.10
	Alcoholic hepatic failure, without coma		K70.40
	Hepatic failure, unspecified, without coma		K72.90
	Alcoholic liver damage, unspecified	571.3	
	Alcoholic liver disease, unspecified		K70.9
	Other specified diseases of liver		K76.89
	Liver disease, unspecified		K76.9
	Portal hypertension	572.3	K76.6
	Hepatorenal syndrome	572.4	K76.7
	Jaundice	782.4	R17.

© 2024 Ilagan-Ying YC et al. *JAMA Network Open*

© 2024 Ilagan-Ying YC et al. *JAMA Network Open*
