# Supplementary material for: Risk Score for Hepatocellular Cancer in Adults Without Viral Hepatitis or Cirrhosis
Source: JAMA Netw Open. 2024 Nov 6;7(11):e2443608. doi: 10.1001/jamanetworkopen.2024.43608 (PMC11541635; doi:10.1001/jamanetworkopen.2024.43608)
Supplement: Supplement 2. — Data Sharing Statement [file jamanetwopen-e2443608-s002.pdf]

Data Sharing Statement

Ilagan-Ying. Risk Score for Hepatocellular Cancer in Adults Without Viral Hepatitis or Cirrhosis. *JAMA Netw Open*. Published November 06, 2024. doi:10.1001/jamanetworkopen.2024.43608

Data
Data available: Yes
Data types: Deidentified participant data
How to access data: VACS@yale.edu
When available: With publication

Supporting Documents
Document types: Statistical/analytic code
How to access documents: VACS@yale.edu
When available: With publication

Additional Information
Who can access the data: researchers whose proposed use of the data has been approved
Types of analyses: replicate findings in an external cohort
Mechanisms of data availability: after approval of a proposal, or with a signed data access agreement
